# Cross-Talk between Signaling Pathways Can Generate Robust Oscillations in Calcium and cAMP

**DOI:** 10.1371/journal.pone.0007189

**Published:** 2009-10-21

**Authors:** Fernando Siso-Nadal, Jeffrey J. Fox, Stéphane A. Laporte, Terence E. Hébert, Peter S. Swain

**Affiliations:** 1 Gene Network Sciences, Cambridge, Massachusetts, United States of America; 2 Centre for Non-linear Dynamics, McGill University, Montreal, Canada; 3 Centre for Applied Mathematics, Cornell University, Ithaca, New York, United States of America; 4 Department of Medicine, McGill University, Montreal, Canada; 5 Department of Pharmacology and Therapeutics, McGill University, Montreal, Canada; 6 Centre for Systems Biology at Edinburgh, University of Edinburgh, Edinburgh, Scotland; Fondazione Telethon, Italy

## Abstract

**Background:**

To control and manipulate cellular signaling, we need to understand cellular strategies for information transfer, integration, and decision-making. A key feature of signal transduction is the generation of only a few intracellular messengers by many extracellular stimuli.

**Methodology/Principal Findings:**

Here we model molecular cross-talk between two classic second messengers, cyclic AMP (cAMP) and calcium, and show that the dynamical complexity of the response of both messengers increases substantially through their interaction. In our model of a non-excitable cell, both cAMP and calcium concentrations can oscillate. If mutually inhibitory, cross-talk between the two second messengers can increase the range of agonist concentrations for which oscillations occur. If mutually activating, cross-talk decreases the oscillation range, but can generate ‘bursting’ oscillations of calcium and may enable better filtering of noise.

**Conclusion:**

We postulate that this increased dynamical complexity allows the cell to encode more information, particularly if both second messengers encode signals. In their native environments, it is unlikely that cells are exposed to one stimulus at a time, and cross-talk may help generate sufficiently complex responses to allow the cell to discriminate between different combinations and concentrations of extracellular agonists.

## Introduction

Intracellular signaling is complex. Few signaling pathways act in isolation, and the coordination of signal transduction is driven by interactions between pathways. Determining the design principles behind this complexity is necessary for understanding and manipulating cellular activity [1]. Cells are unlikely to be exposed to agonists individually, but probably simultaneously receive many extracellular stimuli. We believe that interactions between signaling pathways, so called molecular cross-talk, enables the cell to process and interpret multiple inputs differently in different contexts. For example, a cell exposed to two agonists, each fostering a mutually exclusive response such as growth or apoptosis, must ‘decide’ which signal to follow. Biochemically, this decision is likely to occur with one pathway interacting with and shutting down the activity of the other. In contrast, two weak growth signals detected simultaneously could collectively have an enhanced or synergistic effect, with each pathway reinforcing signaling through the other.

Here we study the effects of cross-talk, or inter-pathway interactions, between two classic second messenger pathways: cAMP and intracellular calcium. Intracellular concentrations of both are modified by the activation of multiple types of receptors, many of which transduce their signals through heterotrimeric G proteins – at the simplest level, G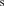 and G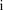 for cAMP and G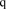 for calcium. Concentrations of cAMP are expected to increase or decrease in response to the detection of an appropriate agonist. Intracellular calcium concentrations can behave similarly, but can also oscillate with a frequency that increases with the concentration of the agonist.

Using mathematical modeling, we investigated whether experimentally demonstrated interactions between the cAMP and calcium responses in non-excitable cells could substantially increase the complexity of the dynamics of their joint response. A gain in complexity increases the potential of the response to encode information relevant to the extracellular environment and so alter decisions affecting cellular behavior. The interaction between calcium and cAMP signals has long been predicted to generate oscillations in cAMP concentrations [2], [3]. Such oscillations have been measured in neurons and agree with simulations using models of excitable cells [4], [5]. Although cross-talk between different calcium pathways has been investigated [6], the effects of multiple simultaneous stimuli and of cross-talk between different second messengers has been little studied, particularly in non-excitable cells.

**Figure 1:**
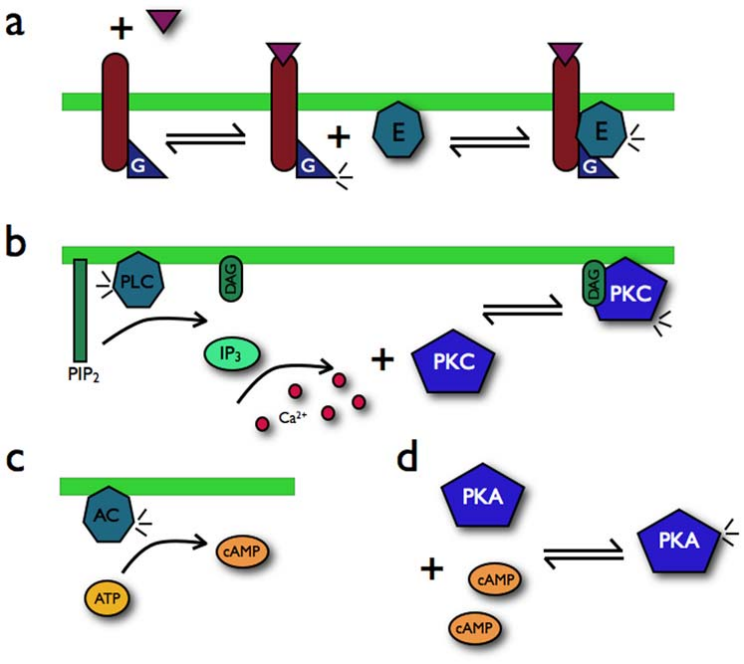
A schematic of the G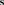, or cAMP, and G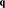, or calcium, pathways. a Agonist (the triangle) binding to receptor activates a G protein, 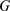, which in turn activates an effector protein, 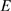. For simplicity, the G protein is always bound to the receptor, and we show explicitly neither G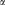 and G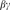 nor the deactivation of the G protein. b The G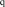 pathway. The effector protein is the 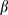 isoform of phospholipase C. When activated, PLC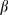 cleaves the phospholipid PIP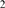 into membrane-bound DAG and cytosolic IP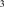. High IP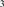 concentrations cause the release of calcium ions from the endoplasmic reticulum. PKC when bound by calcium is recruited to the membrane by DAG and becomes activated. c The G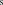 pathway. The effector protein is adenylyl cyclase which synthesizes cAMP from ATP when activated. d The increased concentrations of cytosolic cAMP activate PKA by binding to its inhibitory domain.

The concentration of cytosolic calcium ions increases following the binding of agonist to a G protein-coupled receptor (GPCR) that can activate G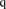. Agonist binding causes the activation of G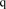 by promoting the exchange of GDP by GTP on the 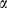 subunit. Activated G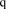 interacts with and activates phospholipase C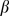 (PLC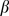) which, when activated, cleaves the membrane phospholipid phosphatidylinositol 4,5-biphosphate (PIP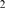). This cleavage generates 1,2-diacylglycerol (DAG), which remains at the membrane, and inositol 1,4,5-trisphosphate (IP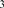), which diffuses into the cytosol (Fig. 1a and 1b). By binding to IP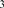-sensitive calcium channels in the endoplasmic reticulum (ER), IP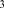 causes the release of calcium from intracellular stores. Among other events, the rise in cytosolic calcium activates classical isoforms of protein kinase C (PKC) which move from the cytosol to the membrane where they can be further activated by DAG. Calcium release often leads to further release of calcium and to calcium oscillations. In our model, calcium oscillations are generated by negative feedback – high concentrations of cytosolic calcium inhibit the release of calcium by IP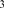 receptors – but positive feedback also occurs because cytosolic calcium increases the rate at which PLC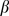 catalyzes the production of IP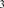.

The concentration of cytosolic cAMP increases through the binding of agonist to GPCRs that activate G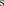. Activated G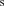 interacts with and activates different isoforms of adenylyl cyclase (AC). Activated AC converts ATP to cAMP and pyrophosphate (Fig. 1a and 1c). As cytosolic cAMP concentrations rise, cAMP binds cooperatively to the two regulatory domains of protein kinase A (PKA) causing the release of its two catalytic subunits (Fig. 1d). PKA further transduces cAMP by phosphorylating many specific target proteins.

We modeled the interactions between cAMP and calcium using non-linear differential equations (Methods). Our model is generic and therefore could form the basis of models for many different cell types and signaling pathways. We focus on predicting qualitative consequences from interactions between cAMP and calcium signaling. Many different types of activated GPCRs can lead to the activation of PLC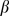 or AC. We wish to focus on the potential effects of several agonists present simultaneously and, rather than describe the activation of each type of receptor, consider the concentrations of activated PLC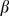 and AC as inputs to our model because it is at these enzymes that the different pathways first converge. Although we expect our results to be qualitatively correct, we do not expect to be able to make quantitative comparisons with experimental data without further specializing the model.

The calcium and cAMP pathways are known to interact with each other in a variety of different ways and cell types. Calcium can influence cAMP signaling directly through modulating the activity of some isoforms of AC and phosphodiesterases [7] and indirectly through PKC: PKC can phosphorylate and so desensitize G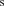-coupled receptors [8], and PKC can interact with some AC isoforms to enhance their enzymatic activity and increase cytosolic cAMP [9]. Alternatively, cAMP can influence calcium signaling through PKA: PKA can sensitize IP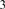 receptors on the ER increasing the release of calcium into the cytosol [10], [11], PKA can increase the activity of calcium ATPases which pump calcium back into the ER [12], PKA can phosphorylate and modulate the activity of receptors coupled to both G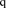 and G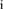 [13], and PKA can phosphorylate and inhibit PLC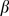 [10], [14], [15], [16], [17], [18]. In addition, elevated cAMP can activate a novel PLC isoform [19], and some GPCRs activate G proteins whose G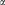 sub-unit modulates AC, but whose G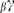 sub-units modulate PLC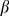 and vice versa [20].

For simplicity, we let calcium affect cAMP concentrations through the action of PKC and let cAMP affect calcium concentrations through the action of PKA. We will consider mutual inhibition, with each pathway inhibiting the other, or mutual activation, with each pathway activating the other. We expect each the nature of the cross-talk to vary between cell types.

**Figure 2:**
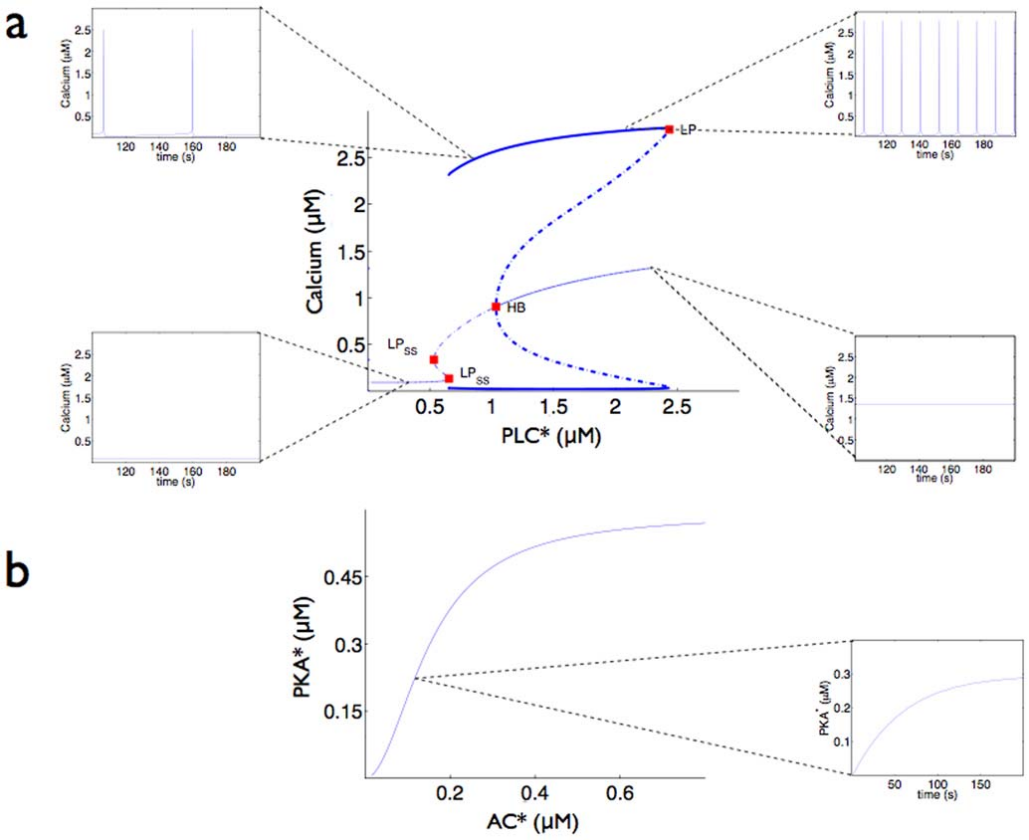
The dynamics of the G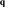 pathway (upper panels) and the G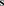 pathway (lower panels). a A bifurcation diagram for the G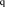 pathway showing the nature of the calcium response for differing concentrations of activated PLC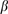. Thin lines indicate a stable steady-state concentration of calcium; thick lines indicate that calcium concentrations oscillate. The upper thick line marks the maximum concentration that calcium reaches during one oscillation; the lower thick line marks the minimum concentration reached. Dotted lines indicate unstable attractors and red squares show bifurcation points. The label HB indicates a Hopf bifurcation; LP indicates a saddle-node bifurcation. The frequency of calcium oscillations ranges from effectively zero Hz when the concentration of PKC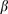 is near 0.65 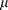
M and to approximately 0.12 Hz (a period of around 8 s) when the concentration of PKC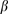 is near 2.4 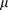
M. Example time courses of calcium concentration illustrating the appearance and disappearance of oscillations are shown at different points in the bifurcation diagram: at activated PLC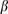 concentrations of 0.3 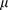
M, 0.9 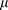
M, 2.0 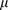
M, and 2.5 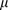
M. b A bifurcation diagram for the G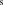 pathway showing stable steady-state concentrations of activated PKA for differing concentrations of activated AC. cAMP concentrations behave similarly and appear linear for the AC concentrations shown. In the example time course at 0.15 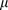
M concentration of activated AC, activated PKA increases monotonically with time while agonists are present. The concentration of cAMP behaves similarly.

We will often use bifurcation diagrams to display qualitative changes in the dynamics of cytosolic calcium as the stimuli to either the calcium or cAMP pathways change. A bifurcation diagram shows the changes in the long-time dynamics of a system as a parameter of the system varies. For example, we will show the dynamics of calcium as a function of the concentration of activity of PLC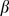 (Fig. 2a). If calcium reaches a steady-state concentration for a range of concentrations of activated PLC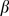, we show these steady-states as a thin line on the bifurcation diagram, with PLC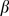 concentrations plotted on the 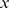-axis. If the system is bistable for a range of concentrations of PLC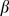, two distinct calcium concentrations are possible at steady-state and the particular concentration reached depends on the condition of the system when the agonist is applied. We show these two concentrations as two thin lines that exist for the same range of PLC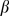 concentrations. We indicate an unstable steady-state, which the system can never reach, as a thin dashed line. If calcium oscillates for a range of concentrations of PLC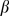, we use two thick lines, one for the maximum and one for the minimum concentration of calcium reached during one oscillation. Concentrations of PLC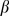 at which there is a transition between two qualitatively different dynamical behaviors, such as steady-state and oscillatory dynamics, are called bifurcation points. Bifurcation diagrams thus succinctly display the general behavior of a dynamical system [21], [22]. We do not expect the form of the bifurcation diagram to alter for small changes in the parameters of the model.

## Results

### Individual pathways with no cross-talk

The dynamics of intracellular calcium are characterized by oscillations (Fig. 2a). Calcium oscillations are generated by delayed negative feedback: high concentrations of cytosolic calcium inhibit calcium release through IP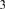 receptors on the ER [23]. Oscillations occur when the concentration of agonists is within a range that corresponds to specific concentrations of activated PLC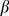. Weak stimuli do not produce enough IP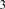 to cause sufficient calcium release from the IP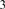 receptors; strong stimuli result in high cytosolic calcium concentrations and inhibit IP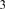 receptors. For intermediate stimuli, the frequency of calcium oscillations increases monotonically as the concentration of the input to the pathway increases.

For activated PLC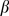 of around 1.1 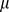
M and higher, the system can either tend to a steady-state, with a constant concentration of calcium, or undergo oscillations in the concentration of calcium: there is a bistability between a steady-state and a limit cycle. The particular dynamics will depend on the initial state of the system when the agonist was applied. Once activated PLC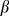 concentrations are high enough, this bistability disappears, and large amplitude oscillations of calcium are lost near the bifurcation point (Fig. 2a): the system tends once again to steady-state.

The dynamics of intracellular cAMP are characterized by steady-states. As the concentration of agonists promoting cAMP production increases so do concentrations of cytosolic cAMP and activated PKA. cAMP concentrations do not oscillate (Fig. 2b).

### Inter-pathway interactions and cross-talk

Interactions between cAMP and calcium signaling can: (i) cause oscillations in both second messengers; (ii) create a bistability between two steady-states; and (iii) generate oscillations with complex waveforms, so-called ‘bursting’ oscillations, through period-doubling bifurcations.

**Figure 3:**
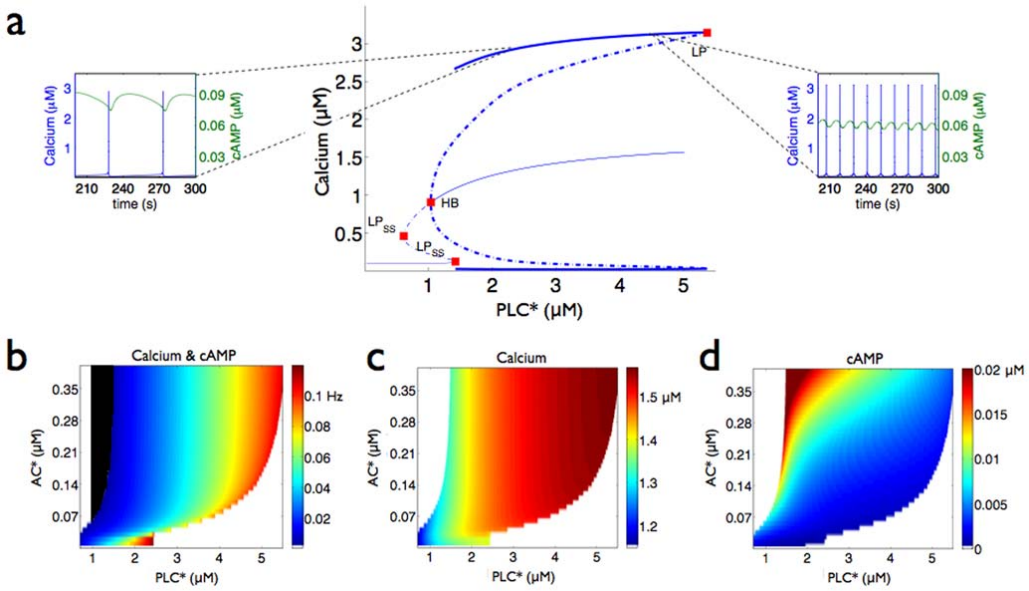
The response of the system with negative or mutually inhibiting interactions between cAMP and calcium. a A bifurcation diagram for calcium as a function of activated PLC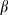 when activated AC is at 0.25 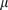
M. Symbols are the same as Fig. 2. Example time courses show oscillations in both calcium and cAMP when activated PLC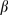 is at 2.1 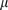
M or 4.5 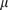
M. The frequency of the oscillations increases with activated PLC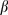 and a phase lag appears between cAMP and calcium. b The frequency of the cAMP and calcium oscillations as a function of both activated AC and PLC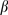 concentrations. A region where the system has two stable steady-states is shaded black. c The amplitude of the calcium oscillations for the same range of activated AC and PLC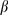. d The amplitude of the cAMP oscillations. We model only global levels of second messengers. Local changes at distinct sub-cellular sites may be much higher.

Negative or mutually inhibitory interactions at least double the range of concentrations of activated PLC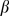 that can generate calcium oscillations (Fig. 3a and 3b). However, they can also prevent oscillations for concentrations of activated PLC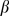 that would normally generate oscillations in the absence of molecular cross-talk. Thus such interactions modulate the sensitivity of the system. The onset and offset concentrations of activated PLC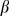 at which oscillations start and stop are increasing functions of activated AC, but the offset concentrations increase more rapidly, and the range of activated PLC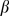 generating oscillations widens.

Oscillations in calcium are transmitted to oscillations in cAMP, whose oscillations in turn modulate those of calcium. Consequently, both second messengers oscillate with the same frequency. At low frequencies, cAMP oscillations are approximately synchronized with calcium oscillations (Fig. 3a), but at higher frequencies there is a significant phase lag (Fig. 3a). The amplitudes of both oscillations are different and change with changes in the stimuli to the system (Fig. 3c and Fig. 3d).

Negative interactions broaden the range of concentrations of activated PLC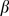 for which calcium oscillations are dominated by a particular frequency (Fig. 3b). Consequently, if we consider signaling only through the frequency of calcium oscillations, negative interactions and exposure to cAMP-generating ligands increases the robustness of signaling to fluctuations in the activity and concentrations of upstream components.

The bifurcation diagram (Fig. 3a) is similar to the bifurcation diagram for calcium signaling in the absence of molecular cross-talk (Fig. 2a). As concentrations of activated PLC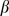 increase, the system passes from a stable steady-state (constant calcium concentrations) to a stable limit cycle (oscillating calcium concentrations) and then bifurcates again to another stable steady-state. There is also, however, a bistability not present in Fig. 2a. For concentrations of activated PLC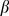 near 1 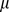
M, two stable steady-states co-exist (Fig. 3a and 3b): one has high concentrations of cytosolic calcium of around 1 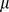
M, corresponding to strong activation of PKC and more modest activation of PKA, and the other has low concentrations of cytosolic calcium and corresponds to reduced activation of PKC and more strong activation of PKA. For two mutually exclusive sets of initial conditions, the system will tend to one of these steady-states if the concentration of activated PLC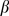 lies in the bistable region.

**Figure 4:**
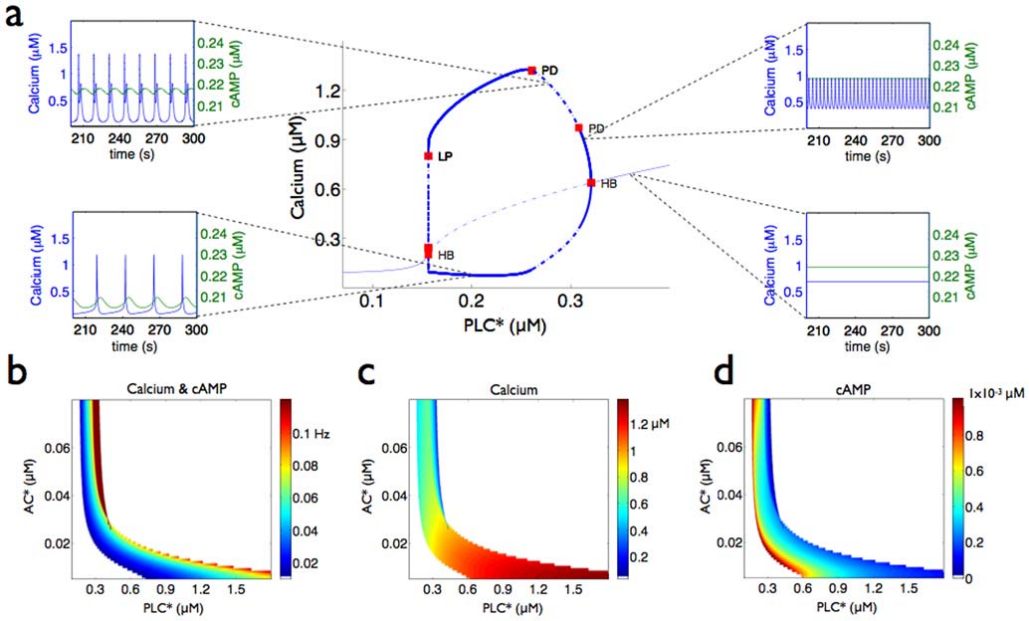
The response of the system with positive or mutually activating interactions between cAMP and calcium. a A bifurcation diagram for calcium as a function of activated PLC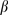 when activated AC is at 0.17 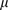
M. Symbols are the same as Fig. 2. PD indicates a period-doubling bifurcation. Example time courses of calcium and cAMP are shown when activated PLC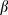 is at 0.2 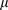
M, 0.28 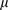
M, 0.31 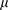
M, and 0.36 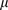
M. Bursting oscillations appear for intermediate activated PLC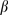 concentrations. The amplitude of the oscillations increases and decreases as activated PLC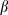 increases, and oscillations gradually disappear. b The frequency of the cAMP and calcium oscillations as a function of both activated AC and PLC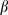 concentrations. c The amplitude of the calcium oscillations. d The amplitude of the cAMP oscillations.

With positive or mutually activating inter-pathway interactions, the range of activated PLC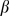 that generates calcium oscillations decreases and more complex oscillations can occur through period-doubling bifurcations (Fig. 4a and 4b). cAMP oscillates with the same frequency as the concentration of calcium (Fig. 4c and 4d) and tends to phase lag at higher frequencies. As concentrations of activated PLC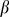 increase, the concentration of cytosolic calcium reaches a steady-state, then undergoes oscillations which can bifurcate into ‘bursting’ oscillations before again reaching another steady-state at higher concentrations of activated PLC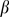 where, contrary to the non-interacting case, the amplitude of the oscillations steadily decrease to zero (Fig. 4a). Increasing concentrations of AC increase the sensitivity to calcium by reducing the concentration of activated PLC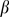 at which oscillations start. The positive interactions not only ‘prime’ the system for release of calcium from the ER, but also allow inhibition of IP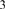 receptors to occur at lower concentrations of activated PLC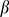 by promoting higher cytosolic calcium concentrations. The higher concentrations of IP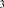 created by positive interactions can cause calcium to be released from the ER in burst-like oscillations. These bursts generate two or more substantial peaks to the waveform of the calcium oscillations (Fig. 4a).

Bursting oscillations in the concentration of calcium have been observed in hepatocytes in response to elevated cytosolic cAMP concentrations and to stimulation by some agonists [24]. Several mechanisms for the generation of such oscillations have been proposed, and all have focused on interactions within the calcium pathway [25], [26], [27], [28], [29]. Although our model is generic and is not intended to accurately describe a hepatocyte per se, it indicates that positive interactions with a background level of activity of the cAMP pathway is another possible explanation of these observations.

Positive interactions may also enable the signaling network to better filter fluctuations in agonist concentrations. The same frequency of calcium oscillations can be achieved by a high concentrations of agonist for G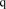-coupled receptors for the non-interacting system or by low concentrations of agonist for both G
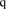
- and G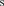-coupled receptors when the pathways interact (Fig. 4b). If the mean concentration of input agonist is low, fluctuations could still drive concentrations of activated PLC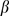 high enough to cause temporary, erroneous activation of downstream proteins. By allowing the frequency of calcium oscillations to encode the presence of two signals, positive inter-pathway interactions reduce such erroneous signaling because the two agonists will rarely both fluctuate simultaneously to high concentrations, even though low levels of agonist are more stochastic. For example, standard simulation techniques show that two stochastic processes with mean 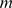, say, both simultaneously cross an arbitrary threshold of 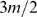 one third less often than a stochastic process with a mean twice as large (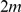) crosses a threshold that is also twice as large (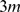), even though the process with mean 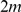 fluctuates less strongly than the process with mean 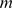 (its coefficient of variation is reduced by approximately 30%).

## Discussion

We have shown that interactions between signaling pathways with different second messengers can generate dynamics similar to those seen in neurons [4] in a generic model of a non-excitable cell, and both cell types may have similar information processing capabilities. Cross-talk can generate cAMP oscillations with the same frequency as calcium oscillations. Such inter-dependent cAMP and calcium oscillations have been observed in non-excitable cells, both in pancreatic 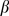 cells [30], [31] and in human embryonic kidney cells [32]. Information can be encoded in the amplitude, phase, waveform, and frequency of these oscillations [33], both for cAMP and calcium. Signaling through oscillations rather than through changes in concentrations at steady-state may provide other advantages for the cell [3], [23]. Information transfer may be less susceptible to corruption by stochastic effects [34]. Oscillations can also enhance signaling efficiency: signals with a mean concentration at steady-state below the threshold of activation of a downstream component might still activate that component if the signals oscillate sufficiently often above threshold [35], and desensitization to stronger input stimuli and large, potentially energetically wasteful, transitions in concentration are both avoided [3].

Within our model cross-talk leads to a number of qualitative experimental predictions. By responding to concentrations of both activated AC and PLC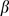, the system gains an extra degree of freedom, with changes to either stimuli generating the same response. For example, decreasing the frequency of cAMP oscillations in Fig. 3b could be achieved by either decreasing the stimulus to the G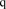 pathway while maintaining the G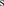 stimulus or by decreasing the stimulus to the G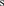 pathway while maintaining the G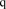 stimulus. We also predict a new region of bistability if inter-pathway interactions are negative (Fig. 3a). For concentrations of activated PLC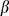 near 1.5 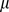
M, the system can act like a toggle switch. Two steady-state concentrations of calcium are possible – one close to zero and the other near 1 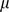
M – and we predict hysteretic, or history-dependent, behavior with the flipping of the switch occurring at different concentrations of activated PLC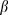 when the concentration of agonists to the G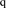 pathway changes from high to low than when it changes from low to high (the stimuli to the G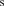 pathway remain unchanged). For a much larger region of activated PLC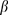 or G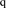 agonist concentrations, there is bistability between a steady-state and an oscillatory response (Fig. 3e), similar to the one present in the G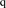 pathway alone. The system can act like an alarm, where a sufficiently large perturbation, for example a change in the concentration of calcium or cAMP, can cause a substantial change in dynamics which will persist once the perturbation has receded. A large perturbation can cause a system with a constant concentration of calcium to begin oscillating with an amplitude of several times the concentration of calcium at steady-state. Similarly, in the bistable region, a system with oscillating calcium and cAMP concentrations can be perturbed in such a way that both oscillations permanently stop.

We have focused on describing the generic behavior generated by cross-talk between the G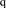 and G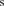 pathways and would expect to adapt our model to describe a particular cell type. Parameters and concentrations of enzymes and receptors will change, but different cell types may also implement cross-talk with different biochemistry or perhaps cross-talk only from one pathway to another and not mutually between both pathways. It is unfortunately experimentally challenging to determine the mechanism generating any detected cross-talk.

Cells are rarely exposed to one stimuli at a time, and yet many signaling pathways converge on only a few second messengers. Considering individual signaling pathways, it is puzzling to understand cAMP and calcium responses and how cells distinguish between different environments characterized by differing combinations of stimuli. For a receptor that activates synthesis of cAMP, steady-state concentrations of cAMP grow monotonically with increased amounts of agonist (Fig. 2b), but will fluctuate because of the inevitable stochasticity present in any biochemical process [36]. From an information theoretic perspective, such a cAMP response may therefore encode little information [37]: with large fluctuations in cAMP, the cell can potentially only discriminate between a concentration of agonist above or below a threshold concentration. Information from concentrations of agonists that change with time, for example in pulses, may be lost. In contrast, receptors that upon binding of agonist increase intracellular concentrations of calcium can cause oscillations in intracellular calcium and so potentially encode more information [33]. For calcium signaling to transmit information at its full capacity without its more complex time-dependent response being redundant, the input agonist concentration must itself have complex time-dependent behavior. Typically, however, agonist concentrations do not: some oscillate but many change from one steady-state concentration to a new higher or lower concentration.

We speculate that cross-talk allows these discrepancies to be understood. A network with inter-pathway cross-talk presumably simultaneously detects multiple signals. The information content of the stimulus to such a signaling network increases substantially. In the simplest scenario, when an agonist concentration is classified only as high or low (by being above or below a threshold concentration), the information content of an input signal of 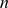 agonists can be 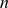 times that of a single agonist [38]. The network's potential to encode information also grows if both cAMP and calcium respond to this signal, particularly because cross-talk increases the dynamical complexity of their response. The cell, in turn, will have more potential to discriminate between different stimuli.

Intracellular calcium with its complex dynamics may provide cells with enough information content to allow discrimination between multiple agonists on its own. Indeed in some cells no evidence of interaction between cAMP and calcium has been found. We would predict that such cells have relatively few types of receptors that activate cAMP or calcium signaling or that such receptors are sequestered in some way to isolate their activity. In general, many agonists modify concentrations of cAMP, and we might therefore expect concentrations of cAMP to also have complex dynamics without any interactions with calcium. The cellular machinery that generates calcium oscillations is elaborate, involving intracellular stores of calcium, shuttling of molecules to and from the plasma membrane, and complex gating schemes of calcium channels. Perhaps rather than create new machinery to generate cAMP oscillations, the pre-existing calcium oscillator may have better served evolution.

For a cell to be able to discriminate between different combinations of stimuli, its response must occur in such a way that the responses from different types of receptors are coordinated and must be of sufficient complexity to encode enough information to allow the discrimination. We postulate that by having many pathways converge on a small number of second messengers, communication occurs between different receptors and have shown here that cross-talk between second messengers potentially generates sufficient complexity in cellular responses to allow discrimination, at least between some combinations of stimuli.

## Methods

Although we consider regulation of both second messengers in general, we will assume that cAMP concentrations are increased through the action of G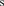 and that calcium concentrations are increased through the action of G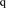. Rather than explicitly model activation of the G proteins, we will consider the concentrations of activated enzymes directly downstream from the G proteins as the inputs to the pathways.

### Modeling calcium signaling

We use the concentration of activated PLC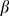 as a measure of the activity of the calcium signaling pathway, which is determined by the total amount of agonists present that modify calcium signaling through PLC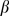. These agonists may bind many different types of GPCR. Our model of calcium signalling is based on that of Meyer and Stryer [39]. Although we ignore several cellular fluxes of calcium, the model generates the experimentally observed calcium dynamics [40], [41] and as such is appropriate for examining the effects of cross-talk with other pathways.

Our model is a system of coupled non-linear differential equations. We assume that the production of IP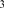 is stimulated by activated PLC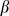 whose activity is enhanced by calcium ions [42]. Letting 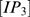 denote the concentration of IP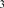, 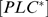 denote the concentration of activated PLC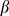, and 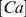 denote the cytosolic concentration of calcium ions, we have
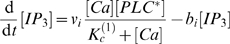
(1)where 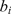 is the turn-over rate of IP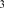, which we assume constant for the time scale of interest; 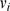 is the maximum rate of synthesis of IP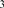 by activated PLC; and 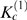 is the concentration of calcium ions at which activated PLC synthesizes IP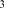 at half its maximum rate. We do not explicitly model PIP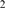. Throughout, the 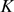 parameters are constant and have units of concentration.

Cytosolic calcium is increased by the release of calcium from the ER by IP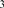 receptors and is decreased by ATPases which pump calcium back into the ER. We assume that the total amount of intracellular calcium – the sum of cytosolic calcium and calcium in the ER – is constant. Calcium is also involved in the activation of PKC. Denoting the concentration of calcium in the ER by 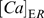 and the concentration of activated PKC by 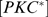, the dynamics of cytosolic calcium is described by
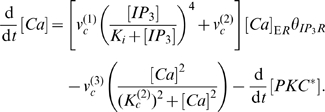
(2)


The opening of IP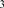 receptors is highly cooperative [43], and we use the Meyer and Stryer model with a Hill coefficient of four [23]. We also allow a small leakage current from the IP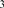 receptors [44] denoted by 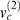. The parameter 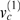 determines the maximum rate that IP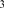 receptors can release calcium ions; 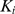 is determined by the affinity of IP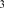 binding to the receptors; 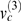 is the maximum rate of activity of the ATPases; and 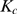 is the concentration of cytosolic calcium ions at which this rate is half-maximal [45]. The fraction of active IP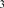 receptors decreases because of the negative feedback caused by high cytosolic calcium concentrations. We model this behavior through the variable 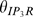


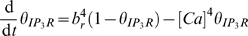
(3)whose steady-state value drops rapidly as a function of calcium [44], and with 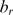 the concentration of calcium ions that makes 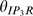 half-maximal.

For the activation of PKC, DAG is necessary [46], and DAG production depends on cleavage of PIP2 by PLC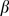, a process enhanced by calcium:
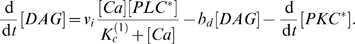
(4)


We assume DAG turns over at a constant rate, 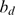, for the time scales we study. We consider the activation of classic isoforms of PKC which require binding to both DAG and calcium. By assuming a fixed total concentration of cytosolic PKC and a two step activation process – PKC first binding calcium and then DAG with the binding of DAG the rate-limiting step, and consequently the intermediate of calcium bound to PKC at quasi-steady-state – we find that the concentration of activated PKC, [PKC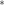], satisfies
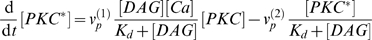
(5)which appears in Eqs. 2 and 4. Here 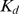 is the ratio of the rate of calcium dissociating from PKC and the rate of DAG binding calcium-bound PKC; 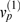 is the rate of calcium binding PKC; and 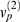 is the product of 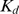 and the rate of dissociation of DAG when bound to PKC.

### Modeling cAMP signaling

We use the concentration of activated AC as a measure of the activity of the cAMP signaling pathway. It is determined by the total amount of agonists present that modify cAMP signaling through AC. These agonists may bind many different types of GPCR, and we do not explicitly model G protein activation.

PKA is activated by increasing concentrations of cytosolic cAMP, denoted 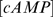. Activated AC (AC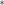) synthesizes cAMP from ATP at a rate 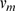, where we assume a constant concentration of cytosolic ATP. Cytosolic cAMP concentrations are constitutively decreased by the enzyme cAMP phosphodiesterase, which converts cAMP to 5′-AMP. Denoting the concentration of activated PKA by 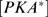, we consequently have that
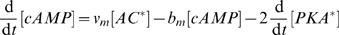
(6)where we have included a constant concentration of phosphodiesterase in the rate 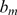. cAMP activates PKA by two molecules of cAMP binding to a single PKA regulatory domain which is then released. PKA is inactivated by the re-binding of free PKA regulatory domains. If we assume a two step process for cAMP binding PKA and that the binding of the second cAMP is rate-limiting so that the intermediate state with just one cAMP bound is at quasi-steady-state, then activated PKA satisfies
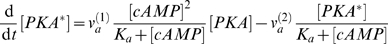
(7)if the total concentration of cytosolic PKA does not change for the time scales we study. Here 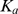 is the ratio of the first cAMP dissociating from PKA and the rate of the second cAMP binding to PKA; 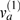 is the rate of association of the first cAMP; and 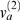 is the product of 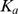 and the rate of dissociation of the second cAMP from PKA. Eq. 7 appears in Eq. 6.

### Parameters

We give the values of the parameters we used in Table 1. We chose some parameters to match an earlier model of calcium signalling [39] (parameters 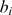, 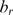, 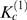, 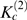, 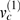, 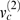, and 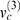). The remaining were chosen to ensure that the concentration of cAMP was of the order of 0.1−0.01 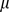
M and that PKA and PKC were sensitive to changes over the entire range of concentrations of their upstream modulators when the pathways were modelled alone (Fig. 2) or together. We used the same parameters when modelling the individual pathways and the negative and positive inter-pathway interactions.

**Table 1:** Values used for the parameters in the model.

Parameter	Value
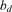	0.5 s 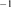
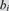	2 s 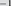
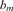	0.15 s 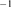
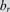	(0.02) 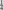 s 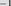
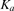	0.01 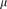 M
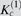	1 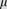 M
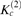	0.15 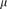 M
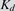	0.2 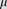 M
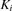	0.4 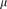 M
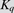	0.003 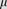 M
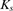	0.02 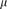 M
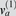	1 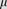 M 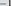 s 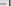
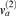	0.01 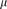 M s 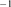
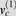	20 s 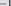
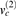	0.01 s 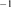
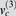	40 s 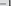
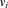	2 s 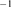
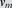	0.1 s 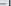
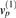	1 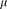 M 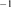 s 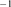
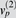	0.7 s 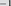
Initial Conditions	Value
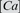	0.1 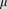 M
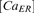	1100 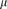 M
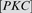	1 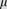 M
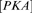	0.59 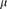 M

### Modeling molecular cross-talk

To model cross-talk between pathways, we assume that an enzyme in one pathway is affected by the activity of the other pathway. We let this enzyme be an allosteric protein with two conformational states, one that strongly promotes signalling and another that only weakly promotes signalling. A negative inter-pathway interaction biases the enzyme to be predominantly in the state that weakly promotes signalling; a positive inter-pathway interaction biases the enzyme to be predominantly in the state that strongly promotes signalling.

We allow PKA to either enhance or inhibit the net activity of PLC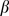 by altering its requirements for calcium ions. We model the affect of PKA on PLC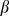 by assuming PLC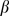 to be allosterically regulated and to have two conformational states, one whose activity is strongly enhanced by calcium and one whose activity is only weakly enhanced. PKA binds to and phosphorylates PLC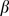 and can enhance IP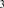 production by binding to the state whose activity is strongly enhanced by calcium or can repress IP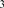 production by binding to the state whose activity is weakly enhanced by calcium. The affinity 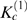 in Eq. 1 and Eq. 4 then becomes
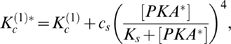
(8)a function of 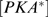. We assume that PKA phosphorylates PLC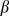 processively on multiple phosphorylation sites and set a Hill coefficient of four to describe their interaction. Decreasing or increasing this Hill coefficient by one little changes the bifurcation diagrams. The parameter 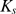 is determined by the affinity of activated PKA for PLC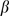. If PKA inhibits the activity of PLC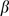 then we set 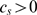, typically to 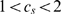; if PKA promotes the activity of PLC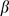 we set 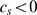, typically to 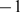. Different values of 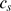 do not qualitatively change the bifurcation behavior except that the negative feedback has to be sufficiently strong (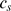 must be above approximately 1) for the bistability between two steady-states to be generated in Fig. 3a.

We allow PKC to enhance or inhibit the enzymatic activity of AC by assuming AC is also an allosteric protein with an active and an inactive state. PKC will bind preferentially to only one of these states. Eq. 6 then becomes
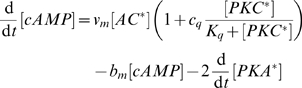
(9)where 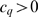, typically 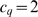, when PKC enhances cAMP production by binding to the active state of AC and 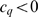, typically 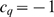, when PKC inhibits cAMP production by binding to the inactive state. The parameter 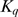 is determined by the affinity of activated PKC to AC.
